# BED ALARMS FOR THE PREVENTION OF FALLS IN HOSPITALS: A THREE-ARM, DISINVESTMENT NONINFERIORITY TRIAL

**DOI:** 10.1093/geroni/igae098.0719

**Published:** 2024-12-31

**Authors:** Terry Haines, Debbie Pu, Kelly Stephen, Ronald Shorr

**Affiliations:** Monash University, Melbourne, Victoria, Australia; Monash University, Melbourne, Victoria, Australia; Monash University, Melbourne, Victoria, Australia; Malcom Randall Veterans Affairs Medical Center, Gainesville, Florida, United States

## Abstract

Bed alarms are commonly used to prevent falls in hospitals. However, the evidence supporting their effectiveness indicates they may be ineffective. Disinvestment from routine but low-value care is important, however carries the risk that unanticipated harms to patients may arise with this change of practice. We undertook a 3-arm, non-inferiority, stepped-wedge randomised trial with concurrent parallel cluster randomised trial to investigate whether reducing the frequency of bed alarm use from >6% of beds to between 3% and 1%, or completely eliminating use of bed alarms had an impact on rates of in-hospital falls. We recruited n=18 wards at hospitals in Melbourne, Australia, and collected data over 10 calendar months. Wards were paired based on type and randomly allocated to month of transition in the stepped-wedge design, and then allocated to either “reduced” or “eliminated” conditions in a two-stage process. Alarm use was monitored through 3x weekly visual audits. Hospital falls data were collected through hospital incident reporting and administrative data systems with weekly auditing from site research assistants. A non-inferiority margin of an increase in rate of falls by 2 falls per 1000 patient days was set in pre-trial stakeholder consultation. The difference between “control (high use of alarms)” and “reduced” conditions was 0.29 falls/1000 patient-days (95%CI: -2.41 to 3.00) favouring the control. The difference between “control” and “eliminated” conditions was 1.13 falls/1000 patient-days (95%CI: -1.58 to 3.83) favouring the control. Non-inferiority of the “reduced” and “eliminated” conditions was not shown, though use of bed alarms was not superior.

